# Acute Aspirin Plus Cilostazol Dual Therapy for Noncardioembolic Stroke Patients Within 48 Hours of Symptom Onset

**DOI:** 10.1161/JAHA.119.012652

**Published:** 2019-07-26

**Authors:** Junya Aoki, Yasuyuki Iguchi, Takao Urabe, Hiroshi Yamagami, Kenichi Todo, Shigeru Fujimoto, Koji Idomari, Nobuyuki Kaneko, Takeshi Iwanaga, Tadashi Terasaki, Ryota Tanaka, Nobuaki Yamamoto, Akira Tsujino, Koichi Nomura, Koji Abe, Masaaki Uno, Yasushi Okada, Hideki Matsuoka, Sen Yamagata, Yasumasa Yamamoto, Toshiro Yonehara, Takeshi Inoue, Yoshiki Yagita, Kazumi Kimura, on behalf of the ADS Investigators, Hidetaka Mitsumura, Yuji Ueno, Masao Watanabe, Yuki Sakamoto, Shuji Arakawa, Yoshinari Nagakane, Ryota Ishibashi, Yuka Terasawa, Koji Fujita, Kenichi Kashihara, Mutsumi Mitomi, Tatsu Nakano, Kensaku Shibazaki, Yoshiki Takao, Yohei Tateishi, Seiji Goto, Yasuhiro Manabe, Naoaki Kanda, Toshihiko Ohashi, Ryo Itabashi, Eisuke Furui, Takaaki Takizawa, Masahiro Minami, Yasuhiro Noguchi, Yoshiyuki Kondo, Tesseki Izumi, Hirokuni Sakima, Yasushi Ueno, Junji Kasuya, Naoki Oba

**Affiliations:** ^1^ Department of Neurological Science Graduate School of Medicine Nippon Medical School Tokyo Japan; ^2^ Department of Stroke Medicine Kawasaki Medical School Okayama Japan; ^3^ Department of Neurology Jikei University School of Medicine Tokyo Japan; ^4^ Department of Neurology Juntendo University Urayasu Hospital Chiba Japan; ^5^ Department of Neurology, Stroke Center Kobe City Medical Center General Hospital Hyogo Japan; ^6^ Department of Cerebrovascular Medicine, Stroke Center Steel Memorial Yawata Hospital Fukuoka Japan; ^7^ Department of Stroke Medicine Okinawa Kyodo Hospital Okinawa Japan; ^8^ Department of Stroke Medicine Okayama Red Cross Hospital Okayama Japan; ^9^ Department of Neurology Japanese Red Cross Kumamoto Hospital Kumamoto Japan; ^10^ Department of Neurology Faculty of Medicine Juntendo University Tokyo Japan; ^11^ Department of Clinical Neurosciences Institute of Biomedical Sciences Tokushima University Tokushima Japan; ^12^ Department of Neurology and Strokology Nagasaki University Hospital Nagasaki Japan; ^13^ Department of Neurology Shioda Hospital Chiba Japan; ^14^ Department of Neurology Okayama University Medical School Okayama Japan; ^15^ Department of Neurosurgery Kawasaki Medical School Okayama Japan; ^16^ Department of Cerebrovascular Medicine and Neurology Clinical Research Institute National Hospital Organization Kyushu Medical Center Fukuoka Japan; ^17^ Department of Cerebrovascular Medicine NHO Kagoshima Medical Center Kagoshima Japan; ^18^ Department of Neurosurgery Kurashiki Central Hospital Okayama Japan; ^19^ Department of Neurology Kyoto Second Red Cross Hospital Kyoto Japan; ^20^ Department of Neurology Stroke Center Saiseikai Kumamoto Hospital Kumamoto Japan; ^21^ Department of Stroke Medicine Kawasaki Medical School General Medical Center Kawasaki Medical School Okayama Japan

**Keywords:** antiplatelet drug, clinical trial, ischemic stroke, noncardioembolic stroke, Clinical Studies, Ischemic Stroke

## Abstract

**Background:**

The aim of the present study was to investigate the efficacy and safety of antiplatelet (aspirin plus cilostazol) dual therapy for patients with noncardioembolic stroke within 48 hours of symptom onset.

**Methods and Results:**

The ADS (Acute Aspirin Plus Cilostazol Dual Therapy for Non‐Cardiogenic Stroke Patients Within 48 Hours of Symptom Onset ) study is an investigator‐initiated, prospective, multicenter (34 hospitals in Japan), randomized, open‐label, and aspirin‐controlled trial. Acute stroke patients with noncardioembolic stroke within 48 hours of onset were studied. The subjects were randomly allocated to combination therapy with aspirin 81 to 200 mg plus cilostazol 200 mg (dual group) and single therapy with aspirin 81 to 200 mg (aspirin group) for 14 days. After the 14 days, all patients took the cilostazol 200 mg for 3 months. A primary efficacy outcome was defined as any one of the following occurring (neurological deterioration, symptomatic stroke recurrence, or transient ischemic attack) within 14 days. A primary safety outcome included intracerebral hemorrhage and subarachnoid hemorrhage. Between May 2011 and June 2017, 1201 patients (796 [66%] men; median age, 69 [61–77] years) randomized 1:1 to either the dual group or the aspirin group were analyzed. Initial National Institutes of Health Stroke Scale score was 2 (1–4) in both groups (*P*=0.830). A primary efficacy outcome was observed in 11% in the dual group and 11% in the aspirin group (*P*=0.853). A primary safety outcome occurred in 2 (0.3%) in the dual group and in 1 (0.2%) in the aspirin group (*P*=0.624).

**Conclusions:**

Dual antiplatelet therapy using cilostazol and aspirin was safe but did not reduce the rate of short‐term neurological worsening.

**Clinical Trial Registration:**

URL: umin.ac.jp/ctr/index/htm. Unique identifier: UMIN000004950.

**Clinical Perspective**

**What Is New?**

- We investigated whether aspirin plus cilostazol dual antiplatelet therapy could reduce the rate of neurological deterioration, stroke recurrence, and transient ischemic attack compared with aspirin alone for noncardioembolic stroke patients within 48 hours of symptom onset.
- Dual antiplatelet therapy using cilostazol and aspirin was safe but did not reduce the rate of short‐term neurological worsening within 14 days.

**What Are the Clinical Implications?**

- Our study findings cannot recommend routine dual antiplatelet therapy using cilostazol and aspirin to reduce the short‐term neurological worsening for patients with acute noncardioembolic stroke.

## Introduction

Stroke is the leading cause of long‐term disability. Beside initial damage, stroke progression results in unfavorable outcomes. The most intensive and safest therapeutic strategies have been investigated for decades. Apart from intravenous thrombolysis and endovascular therapy, aspirin has played a key role by reducing relative risk of stroke recurrence by 20%.^1^ The recent prospective randomized CHANCE (Clopidogrel in High‐Risk Patients With Acute Nondisabling Cerebrovascular Events) and POINT (Platelet‐Oriented Inhibition in New Transient Ischemic Attack [TIA] and Minor Ischemic Stroke) studies found that adding clopidogrel to aspirin decreased neurological worsening in patients with acute noncardioembolic stroke, which subsequently impacted stroke guidelines.^2^, ^3^ However, this regimen may be limited in terms of an increase in the incidence of hemorrhagic complications.

Cilostazol is an antiplatelet agent that has been used to manage stroke. In addition to antiplatelet effects, cilostazol also has pleiotropic effects on neurological function, neurogenesis, neovascularization, and inflammation.^4^, ^5^ The CSPS 2 (Cilostazol for Prevention of Secondary Stroke) trial concluded that cilostazol was somewhat superior to aspirin for preventing secondary stroke with fewer hemorrhagic events.^6^, ^7^ Another multicenter, randomized study (CSPS.com [Cilostazol Stroke Prevention Study for Antiplatelet Combination]) is in progress to determine whether combining cilostazol with aspirin can prevent secondary ischemic stroke more effectively in high‐risk patients.^8^ A trial of cilostazol to treat acute ischemic stroke suggested that it is feasible and comparable to aspirin in terms of effectiveness and safety.^9^ Two trials of symptomatic intracranial arterial stenosis (TOSS [Trial of Cilostazol in Symptomatic Intracranial Arterial Stenosis] and TOSS‐2) showed that cilostazol has the potential to prevent progressive intracranial artery stenosis,^10^, ^11^ but subsequent small pilot studies^12^, ^13^ did not confirm whether cilostazol plus aspirin can improve the clinical outcomes of patients with acute stroke.

Therefore, we conducted a large, multicenter, prospective, and randomized trial, ADS (Acute Aspirin Plus Cilostazol Dual Therapy for Non‐cardiogenic Stroke Patients Within 48 Hours of Symptom Onset), to investigate whether this combination of the dual antiplatelet therapy reduces the rate of neurological deterioration, stroke recurrence, and TIA compared with aspirin alone.

## Methods

Because of ethical restrictions on data protection issues, data that support the findings of this study cannot be made publicly available; however, the data are available to other researchers from the corresponding author upon request when our institutional review boards approve it.

### Study Design

The ADS multicenter prospective, randomized, open‐label aspirin‐controlled study involving 34 institutions in Japan proceeded between May 2011 and June 2017. The institutional review boards at all participating institutions approved the study, which was registered with the Japan Clinical Trials Registry (http://umin.ac.jp/ctr/index/htm), under the number UMIN000004950. The study was organized by a coordinating center at Kawasaki Medical School from May 2011 until June 2014 and then by Nippon Medical School from July 2014 until June 2017. The principal investigator (K.K.) had complete access to all data and was responsible for preparing the manuscript. Clinical data were registered in an online database managed by the Shibasho Corporation (Osaka, Japan).

### Patients

Patients, or a family member as an acceptable surrogate, provided written institutional review board–approved informed consent to participate in this trial. The inclusion criteria comprised age ≥18 years, noncardioembolic stroke presented within 48 hours of symptom onset, neurological deficits with National Institutes of Health Stroke Scale (NIHSS) scores <20, and a premorbid modified Rankin Scale (mRS) score between 0 and 2. The exclusion criteria comprised cardioembolic stroke with a high‐risk source defined by the TOAST (Trial of Org 10172 in Acute Stroke Treatment) criteria,^14^ under medication with antiplatelet agents, cilostazol, aspirin ≥200 mg, clopidogrel, ticlopidine, or any anticoagulants before stroke onset; having undergone or planning to undergo thrombectomy; bleeding or tendency to bleed; pregnant or lactating; congestive heart failure; gastrointestinal ulcers; malignancy within the past 5 years; allergies to salicylic acid or cilostazol; and judged inappropriate for the study by an investigator.

### Randomization

Eligible patients were randomly assigned 1:1 to groups that received either aspirin plus cilostazol (dual group) or aspirin (aspirin group). Randomization was conducted using an online system managed by the Shibasho Corporation. The randomization schedule included a block design by each institution. We assumed that the reduction rates of combined neurological deterioration, symptomatic stroke recurrence, and TIA rates were 15% and 30% in the aspirin and dual groups, respectively; thus, each group required 862 patients (α=5%, 2‐sided; power, 80%). Accounting for possible dropouts, the calculated sample size was 2000 patients. All investigators agreed with the treatment allocation.

The dual group was administered cilostazol (200 mg/day) and aspirin (80–200 mg/day) for 14 days. Thereafter, cilostazol (200 mg) was continued for 3 months after onset. The aspirin group was administered aspirin (80–200 mg/day for 14 days followed by cilostazol (200 mg/day) until 3 months after onset. Because neurological status frequently deteriorated within the acute phase and long‐term dual therapy might lead to significant hemorrhagic complications, the periods of dual therapy were 2 weeks. Concomitant warfarin, clopidogrel, ticlopidine, sarpogrelate, and urokinase were prohibited, whereas argatroban, ozagrel, heparin, edaravone, glycerin, and low‐molecular‐weight dextran were permitted.

### Evaluation

#### Clinical background and characteristics

The following clinical information was obtained: (1) age and sex; (2) premorbid mRS score; (3) onset time and hospital arrival time; (4) vascular risk factors; (5) aspirin and hypertension, hyperlipidemia, and diabetes mellitus drugs before stroke; and (6) previous history of stroke. Vascular risk factors were identified as (1) hypertension, a history of using antihypertensive agents, a systolic blood pressure ≥140 mm Hg, or a diastolic blood pressure ≥90 mm Hg at hospital discharge; (2) diabetes mellitus, the use of hypoglycemics, random glucose level ≥200 mg/dL, or glycosylated hemoglobin ≥6.5% on admission; and (3) hyperlipidemia, the use of antihyperlipidemic agents, or serum cholesterol level >220 mg/dL. Neurological deficits were assessed using the NIHSS score before and at 24 hours, 48 hours, 7 days, 14 days (or at discharge) after admission. NIHSS score was also recorded if clinical symptoms deteriorated or new neurological symptoms appeared. Blood pressure and heart rates were also documented.

Patients were assessed by magnetic resonance imaging and magnetic resonance angiography upon admission and on day 7±3 thereafter, as well as on the day when neurological symptoms deteriorated or on the day when new neurological symptoms occurred where possible. The number, location, size of infarct as well as white matter lesions (deep and subcortical white matter hyperintensity, Fazekas grade), and microbleeds were evaluated by magnetic resonance imaging. Intracranial stenosis and occlusion were assessed by magnetic resonance angiography. Internal carotid artery stenosis and occlusion were evaluated using cervical ultrasonography and conventional cerebral angiography as appropriate. Cardiac function was assessed using electrocardiography, chest radiography, and transthoracic and transesophageal echocardiography as appropriate. Atrial fibrillation upon admission was assessed by ECG and monitored using a Holter ECG during hospitalization.

All patients had baseline blood samples drawn upon admission. The white blood cell count, red blood cell count, hematocrit, hemoglobin, platelets, glutamic‐oxaloacetic transaminase, aspartate aminotransferase, alanine aminotransferase glutamic‐pyruvic transaminase, albumin, lactase dehydrogenase, total bilirubin, glucose, blood urea nitrogen, creatinine, total cholesterol, low‐density lipoprotein cholesterol, high‐density lipoprotein cholesterol, triglyceride, C‐reactive protein, d‐dimer, estimated glomerular filtration rate, hemoglobin A_1c_, insulin, and brain natriuretic peptide were calculated.

At discharge, the final diagnosis was determined. Stroke classification was divided into large‐artery atherosclerotic, lacunar, branch atheromatous disease, other determined infarct (paradoxical embolism, cerebrovascular dissection, aortogenic embolism, others), undetermined infarct, cardioembolic stroke, and TIA. Large‐artery atherosclerosis required a major arterial lesion ≥50%. Branch atheromatous disease was defined as those in the perforating artery territory with infarct diameter ≥1.5 cm without major arterial lesions. At 3 months after onset, mRS was evaluated at each in institution.

### Primary Outcome

A primary efficacy outcome was defined as any one of the following occurring within 14 days of onset: neurological deterioration, symptomatic stroke recurrence, and TIA. Neurological deterioration was defined as positive when NIHSS scores increased by 2 or more points. A primary safety outcome included intracerebral hemorrhage and subarachnoid hemorrhage within 14 days of onset.

### Secondary Outcome

A secondary outcome included (1) mRS ≤1 at 3 months after onset; (2) major adverse cardiovascular events included stroke recurrence, myocardial infarction, cardiovascular death, and life‐threatening bleeding within 14 days and within 3 months; (3) intracranial and extracranial hemorrhages within 14 days and within 3 months; and (4) adverse events including gastrointestinal symptom, headache, and palpitation within 14 days and within 3 months of onset. A serious adverse event was defined as an event caused by treatment that met any of the following criteria: fatal, life threatening, required or prolonged inpatient hospitalization, resulted in persistent or significant disability/incapacity, or a significant medical event in the investigator's judgment.

### Statistical Analysis

We compared clinical and radiological backgrounds, as well as primary and secondary outcomes between the dual group and the aspirin group. Next, the cumulative events prevalence was estimated and plotted by use of Kaplan–Meier analysis. The prevalence of the primary efficacy outcome per day was calculated in each group with the log‐rank test. We also calculated the hazard ratios (95% CI) of dual therapy to aspirin therapy for prevalence of the primary and secondary efficacy outcomes, including subgroup analysis using the parameters of age, premorbid mRS, NIHSS score, onset to admission, concomitant argatroban/heparin therapy, microbleeds, deep subcortical white matter hyperintensities, and symptomatic major artery stenosis/occlusion. We used above‐mentioned parameters because they had been generally considered to be associated with clinical outcome. An investigator at the central coordinating center (J.A.) statistically analyzed the data. Differences in continuous variables were analyzed using Mann–Whitney *U* tests, and differences in categorical variables were analyzed using Fisher exact tests and Pearson chi‐square tests. Data are presented as medians (interquartile ranges) or frequencies (percentages). All data were statistically analyzed using SPSS version 22 (SPSS Japan, Inc., Tokyo, Japan). Differences were considered statistically significant at *P*<0.05.

## Results

### Patient Flow

**Figure 1:**
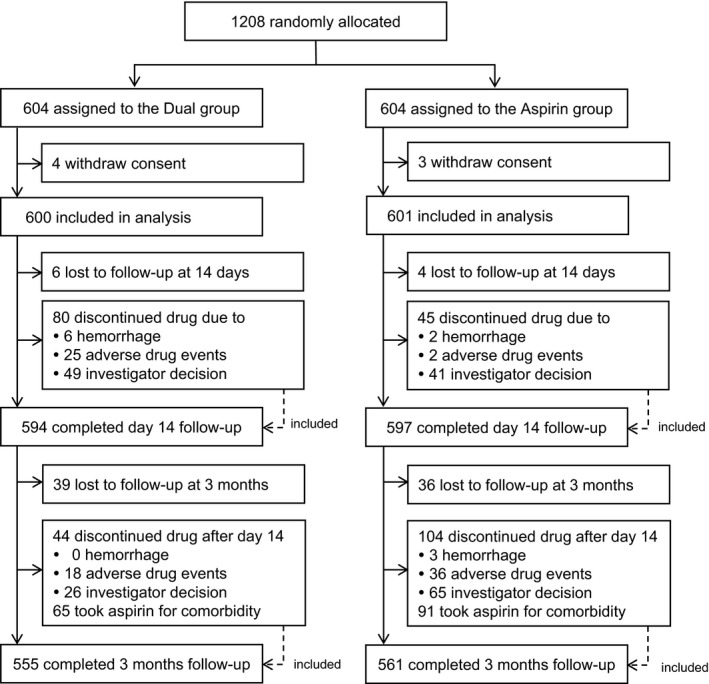
Trial profile (intention‐to‐treat analysis).

Between February 2011 and Mach 2017, 1208 patients were enrolled into the ADS. The trial was halted in March 2017 because of slow enrollment, reaching only 60% of the anticipated number of patients within 6 years. Seven patients withdrew consent after the study started; thus, data derived from 1201 patients were analyzed. Figure 1 shows the detailed flowchart. As shown, 594 (99%) patients in the dual group and the 597 (99%) in the aspirin group completed the day 14 assessment. After discharge, 555 (93%) in the dual group and 561 (93%) in the aspirin group received the final evaluation at 3 months.

### Drug Discontinuation

Drug discontinuation within 14 days was observed frequently in the dual group, and, conversely, discontinuation after 14 days and within 3 months was frequent in the aspirin group. Within 14 days, 49 patients in the dual group and 41 patients in the aspirin group discontinued the allocated drugs because of the investigator's decision: (1) administration of anticoagulant therapy in 35 in the dual group and in 24 in the aspirin group (attributable to new detection of atrial fibrillation, deep vein thrombosis, etc); (2) cardiac dysfunction in 3 in the dual group; (3) neurological deterioration and/or recurrent stroke in 1 in the dual group and in 5 in the aspirin group; (4) to prevent hemorrhagic events in 2 in both groups (for invasive procedures); (5) early discharge in 2 in the dual group and in 1 in the aspirin group; (6) intracranial aneurysm in 1 in the aspirin group; and (7) others in 6 in the dual group and in 8 in the aspirin groups. After 14 days to 3 months, 26 patients in the dual group and 65 patients in the aspirin group discontinued because of (1) anticoagulant therapy in 6 in the dual group and in 5 in the aspirin group; (2) cardiac dysfunction in 3 in the dual group and in 4 in the aspirin group; (3) neurological deterioration and/or recurrent stroke in 2 in the dual group and in 8 in the aspirin group; (4) to prevent hemorrhagic events in 4 in the dual group and in 5 in the aspirin group; (5) intracranial aneurysm in 1 in the dual group; (6) aspirin therapy in 4 in the dual group and in 23 in the aspirin group; (7) clopidogrel addition in 2 in the dual group and in 3 in the aspirin group; (8) no antiplatelet therapy in 3 in the dual group and in 1 in the aspirin group; (9) referral facility decision in 2 in the aspirin group; (10) patient preference in 6 in the aspirin group; and (11) others in 1 in the dual group and in 8 in the aspirin group.

### Clinical Characteristics and Image Findings

Patient clinical background at the time of randomization was balanced except for the premorbid activity (Table 1). The prevalence of mRS 0 before stroke onset was less frequent in the dual group compared with the aspirin group (*P*=0.054). Regarding the acute treatment, argatroban and heparin were frequently administered in the aspirin group (*P*=0.016, and 0.037, respectively). The dual group spent less time in the hospital than the aspirin group (*P*=0.028). Table 2 shows the initial magnetic resonance imaging and echographic findings as well as those on day 7. Over 70% of patients had a single infarct of ≤1.5 cm. Initial magnetic resonance imaging parameters did not significantly differ between the two groups. However, the prevalence of infarct enlargement tended to be lower in the dual group on day 7, but the difference did not reach significance (12% versus 15%; *P*=0.146). Tables S1 through S3 show other comparative data between the two groups.

**Table 1:** Clinical Characteristics Between the Dual Group and the Aspirin Group

Variables	Dual Group (n=600)	Aspirin Group (n=601)	*P* Value
Age, y, median (IQR)	69 (60–77)	69 (61–78)	0.380
Male sex, n (%)	398 (66)	398 (66)	0.999
Premorbid modified Rankin Scale 0, n (%)	505 (84)	529 (88)	0.054
National Institutes of Health Stroke Scale score, median (IQR)	2 (1–4)	2 (1–4)	0.830
Onset to admission, hour, median (IQR)	10.1 (4.6–20.0)	11.5 (4.8–20.9)	0.213
Vascular risk factor, n (%)			
Hypertension	456 (76)	469 (78)	0.492
Diabetes mellitus	192 (32)	190 (32)	0.901
Hyperlipidemia	276 (46)	291 (49)	0.419
Aspirin therapy before onset, n (%)	58 (10)	61 (10)	0.847
Statin therapy before onset, n (%)	84 (14)	92 (15)	0.568
Past history, n (%)			
Ischemic stroke	53 (9)	62 (10)	0.433
Intracerebral hemorrhage	8 (1)	8 (1)	0.999
Systolic blood pressure, mm Hg, median (IQR)	162 (146–180)	161 (145–180)	0.486
Diastolic blood pressure, mm Hg, median (IQR)	90 (80–102)	90 (80–101)	0.860
Aspirin dosage, n (%)			
81 to 100 mg	127 (21)	108 (18)	0.167
162 to 200 mg	469 (79)	491 (82)	
Concomitant therapy, n (%)			
Argatroban	143 (24)	181 (30)	0.016
Heparin	40 (7)	61 (10)	0.037
Statin	341 (57)	343 (57)	0.999
Day of hospitalization, median (IQR)	14.5 (10.4–21.4)	15.6 (10.41–24.7)	0.028
Major etiologies of stroke on discharge, n (%)			
Large artery atherosclerosis	75 (13)	92 (15)	0.182
Lacunae	276 (46)	256 (43)	0.245
Branch atheromatous disease	80 (13)	93 (16)	0.324

IQR indicates interquartile range.

**Table 2:** Magnetic Resonance Findings Between the Dual Group and the Aspirin Group

Variables	Dual Group (n=600)	Aspirin Group (n=601)	*P* Value
Initial magnetic resonance			
Number of infarcts^a^			
None	25 (4)	20 (3)	
Single	459 (77)	457 (76)	0.785
Multiple	113 (19)	123 (21)	0.514
Size of infarcts^b^			
≤1.5 cm	421 (74)	419 (72)	0.643
1.5–3.0 cm	114 (20)	120 (21)	0.770
≥3.0 cm	37 (7)	41 (7)	0.726
Microbleeds^c^	173 (29)	163 (27)	0.562
Deep and subcortical white matter hyperintensity^d^	424 (71)	434 (73)	0.608
Intracranial arterial stenosis^e^			
Moderate	41 (7)	32 (5)	0.280
Severe	20 (3)	20 (3)	0.999
Occlusion	41 (7)	49 (8)	0.443
Significant carotid stenosis/occlusion^f^	58 (10)	60 (10)	0.923
Magnetic resonance on day 7^g^			
Infarct enlargement	71 (12)	87 (15)	0.146

Data are shown in number and percentage.

a Data on 3 patients in the dual group and 1 patient in the aspirin group were not available.

b Eighteen in the dual group and 21 in the aspirin group.

c Three in the dual group and 4 in the aspirin group.

d Three in the dual group and 2 in the aspirin group.

e Three in both groups.

f Six in the dual group and 5 in the aspirin group.

g Twenty‐two in the dual group and 31 in the aspirin group were not available.

### Primary Efficacy and Safety Outcomes

**Figure 2:**
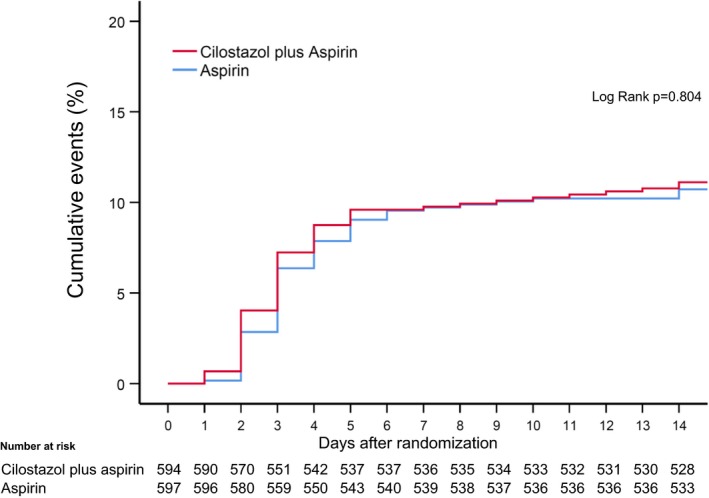
Cumulative incidence of primary efficacy outcome.

**Figure 3:**
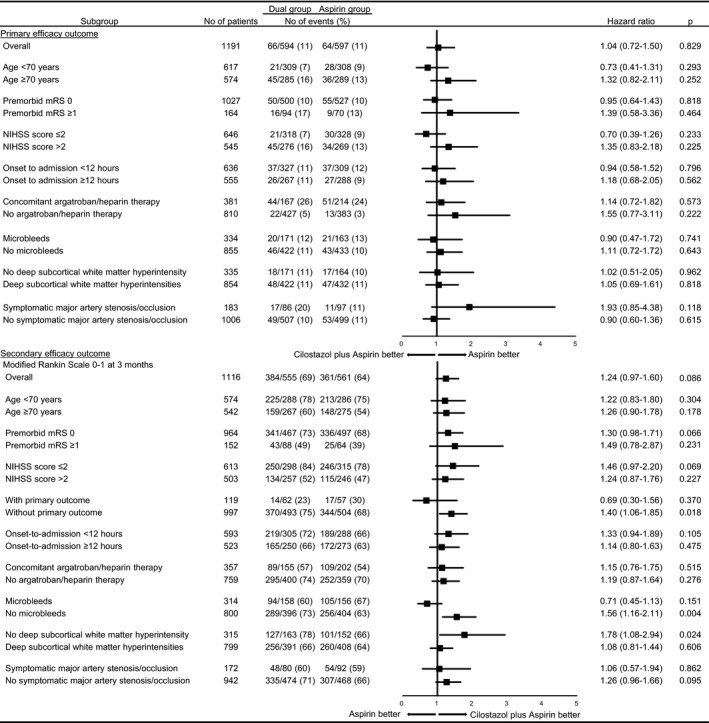
Primary and secondary outcomes, according to subgroups. NIHSS indicates National Institutes of Health Stroke Scale.

The prevalence of the primary efficacy, defined as neurological deterioration, symptomatic stroke recurrence, or TIA occurring within 14 days of onset was similar between the 2 groups: 11% in the dual group and 11% in the aspirin group (*P*=0.853).There was no difference in the timing of the primary efficacy outcome (Figure 2) or in the contents of the primary efficacy outcomes between the dual and the aspirin groups (Table 3). Regarding the primary safety outcome, the dual therapy did not increase the incidence of the intracerebral hemorrhage and subarachnoid hemorrhage complications compared with the aspirin group (2 [0.3%] versus 1 [0.2%]; *P*=0.624). Only 1 patient in the dual group had symptomatic intracerebral hemorrhage, and the others were asymptomatic. A subanalysis based on the specific parameters also demonstrated a similarity in the rate of the primary efficacy outcome (Figure 3).

**Table 3:** Major Outcomes Between the Dual Group and the Aspirin Group

Variables	Dual Group (n=600)	Aspirin Group (n=601)	*P* Value
Primary efficacy outcome, n (%)^a^			
Neurological deterioration	63 (11)	58 (10)	0.632
Recurrent ischemic stroke	6 (1)	8 (1)	0.789
Transient ischemic attack	1 (0.2)	1 (0.2)	0.999
Primary safety outcome, n (%)^a^			
Intracerebral hemorrhage	1 (0.2)	1 (0.2)	0.999
Subarachnoid hemorrhage	1 (0.2)	0 (0)	0.499
Secondary efficacy outcome, n (%)			
Modified Rankin Scale score ≤1 at 3 mo^b^	384 (69)	361 (64)	0.087
Secondary safety outcomes, n (%) within 14 d^a^			
Major adverse cardiovascular event	12 (2)	12 (2)	0.999
Extracranial hemorrhage			
Gastrointestinal	2 (0.3)	2 (0.3)	0.999
Urinary	1 (0.2)	0 (0)	0.499
Others	2 (0.3)	2 (0.3)	0.999
Serious	2 (0.3)	1 (0.2)	0.624
Adverse event			
Gastrointestinal	2 (0.3)	0 (0)	0.249
Headache	32 (5)	7 (1)	<0.001
Palpitation	14 (2)	7 (1)	0.130
Others	19 (3)	6 (1)	0.009
Serious	0 (0)	1 (0.2)	0.999

a Six patients in the dual group and 4 patients in the aspirin group were excluded.

b Forty‐five patients in the dual group and 40 patients in the aspirin group were excluded.

### Secondary Efficacy Outcomes

Regarding the secondary outcome, the prevalence of mRS 0 to 1 at 3 months was equal between the dual group and the aspirin group (69% versus 64%, *P*=0.087; and hazard ratio, 1.24 [0.97–1.60], *P*=0.086). The subanalysis findings on detail parameters in terms of the mRS ≤1 are shown in Figure 3. Patients without a primary outcome, microbleeds, or white matter change were those who had the benefit of dual therapy (*P*=0.018, 0.004, 0.024, respectively). Besides the mRS, other secondary efficacy outcomes were similar between the groups. The prevalence of major adverse cardiovascular events within 14 days was 2% in both group (*P*=0.999), and within 3 months was 4% in the dual group and 5% in the aspirin group (*P*=0.775; Table S4). The prevalence of combined neurological deterioration, recurrence of ischemic stroke, and TIA within 3 months was also similar, at 13% in both group (*P*=0.999).

### Secondary Safety Outcomes and Adverse Events

Beyond 14 days to 3 months, 2 patients in the aspirin group had intracranial hemorrhage (1 intracerebral hemorrhage, and 1 subdural hematoma) (*P*=0.500). Thus, the number of patients who had intracranial hemorrhage within 3 months was calculated as 2 (0.4%) in the dual group and 3 (1%) in the aspirin group (*P*=0.999). The details are presented in Table 3 and Table S4. Although extracranial hemorrhages and adverse events were the major reasons for drug discontinuation, not all led to drug discontinuation. Extracranial hemorrhage within 14 days was observed in 5 (1%) in the dual group and 4 (1%) in the aspirin group (*P*=0.752). From 14 days to 3 months, only 6 (1%) in the aspirin group (*P*=0.031) had extracranial hemorrhage, which resulted in similar rates of extracranial hemorrhage within 3 months: 5 (1%) in the dual group and 10 (2%) in the aspirin group (*P*=0.299).

Adverse events were correlated with the timing of cilostazol administration. Within 14 days, 67 (11%) events occurred in the dual group and 20 (3%) in the aspirin group (*P*<0.001). Between 14 days and 3 months, 30 (5%) in the dual group and 54 (10%) in the aspirin group had adverse events (*P*=0.009). Within 14 days, headache developed in 32 (5%) in the dual group and in 7 (1%) in the aspirin group (*P*<0.001), and others (mainly asymptomatic tachycardia) were seen in 19 (3%) in the dual group and 6 (1%) in the aspirin group (*P*=0.009). However, these differences disappeared at 3 months: 6% versus 4% (*P*=0.137) and 6% versus 5% (*P*=0.437), respectively.

## Discussion

The addition of cilostazol to aspirin did not decrease the prevalence of combined neurological deterioration, stroke recurrence, and TIA events within 14 days. Given this fact, we cannot recommend dual antiplatelet therapy using cilostazol and aspirin in preventing short‐term neurological worsening in patients with acute stroke.

A hypothesized or proven theory of stoke deterioration in noncardioembolic stroke covers a lot of ground. Failure of collateral blood flow, thrombus propagation, cerebral edema, excitotoxicity, and inflammation have been reported.^15^ One of the goals of antiplatelet therapy is to diminish thrombus propagation by suppressing platelet aggregation. Cilostazol inhibits platelet aggregation by inhibiting phosphodiesterase 3 and increasing cAMP concentrations.^16^ The effectiveness of cilostazol as a mono–antiplatelet therapy has been reported in preventing recurrent ischemic stroke, decreasing the rate of plaque generation after carotid stenting, and suppressing the peripheral artery atherosclerotic progression.^6^, ^17^, ^18^ However, our ADS did not provide an additional efficacy of dual antiplatelet therapy using cilostazol and aspirin. One explanation may be the clinical backgrounds of our cohort. Although 1 of our inclusion criteria was NIHSS score ≤20, most of our patients had minor, nondisabling neurological deficits. Low rates of large‐artery atherosclerosis and branch atheromatous disease might explain the similarity in the rates of primary outcome events between the 2 groups. In the CHANCE substudy on 481 patients with intracranial artery stenosis, patients had higher rates of recurrent stroke than those without intracranial artery stenosis.^19^ Previous small studies showed the possibility of reduced stenosis progression achieved by combined cilostazol and aspirin therapy.^10^, ^20^ The ongoing CSPS.com may provide the additional safety and efficacy of combining cilostazol with aspirin therapy against intracranial artery stenosis.^8^ Another explanation may be the study design. We permitted a low dose of aspirin therapy below 200 mg before onset and intravenous administration of ozagrel that inhibit thromboxane A2 synthesis after admission as well as anticoagulation therapy^21^, ^22^ because our steering committee had decided to evaluate the additional effects on routine practice provided by the combined cilostazol and aspirin therapy. A small number of patients with large‐artery atherosclerosis and concomitant therapies might reduce the impact of cilostazol on the primary outcome.

We proved the safety of the addition of cilostazol to aspirin in terms of the hemorrhagic events. In our study, the prevalence of both intracranial hemorrhagic and extracranial hemorrhagic complications was similar between the dual group and the aspirin group, including the severity. This is one of the advantages of cilostazol therapy, which is identical to the findings of CSPS 2 (Cilostazol for Prevention of Secondary Stroke) trial.^6^ It has been a critical issue to guarantee safety when antiplatelet therapy is administered. The number and the duration of antiplatelet drugs are related to safety. For example, the risk of severe bleeding doubled in a study of patients with acute cerebral ischemia (TARDIS [Triple Antiplatelets for Reducing Dependency After Ischemic Stroke]) treated with a combination of aspirin, clopidogrel, and dipyridamole for 30 days compared with clopidogrel alone or aspirin plus dipyridamole.^23^ When focusing on the dual antiplatelet therapy with clopidogrel and aspirin, although 14 days’ administration in the CHANCE study showed a preferable finding in safety,^2^ longer administration for 3 months in the POINT study demonstrated 5 major hemorrhagic complications in exchange for the 15 ischemic stroke preventions.^3^ We must consider that cilostazol has adverse events such as headache and palpitation caused by vessel dilatation. However, the effect of these events was not permanent. Neither coronary artery disease nor congestive heart failure had increased in the acute phase. An incremental increase in dose from 50 mg of cilostazol was shown to decrease the adverse events.^6^ This indicates that concerns regarding side effects should not prevent cilostazol administration.

Cilostazol has pleiotropic effects in addition to the antiplatelet effectivity, which inhibits the infarct expansion, maintains motor and special functions, and increases blood flow, and hemodynamic effects by increasing intracellular endothelial nitric oxide synthase.^4,^ 24 Other studies reported the availability of promoting neoangiogenesis, induced by the inhibition of the astrocyte and encouraged pericyte, resulting in increments of the vascular endothelial growth factor receptor 2 concentrate level.^5^, ^25^ Reducing inflammation is reported to enhance clinical efficacy.^26^ Because inflammation mediates atherosclerosis progression, plaque rupture, and thromboembolism, agents that inhibit inflammation might lead to clinical recovery. In our study, although it was the subanalysis finding of the secondary efficacy outcome, patients without a primary efficacy event as well as those without deep white matter change had the beneficial effect of dual therapy. These results are partly in line with a previous pilot study^12^ in which the mean improvement in NIHSS score at day 14 tended to be better among patients who did not deteriorate and were treated with aspirin plus cilostazol, compared with aspirin. Neurological deficits can improve over time, especially when they are mild. However, a definite approach to achieve clinical recovery has not been established.^27^ Further study considering various aspects in addition to the antithrombotic mechanism may be recommended.

This study has several limitations. Patient registration was halted because of slow enrollment. Approximately 14% to 20% of patients discontinued during the study periods, as presented in the results. Another limitation included the design of this study. Contraindications of cilostazol included patients already diagnosed as having heart failure of any severity and hypersensitivity to cilostazol or any component of cilostazol. Therefore, efficacy and safety of combined cilostazol and aspirin therapy for those patients could not be evaluated. Finally, ADS was an open‐label, not a double‐blind study.

In conclusion, dual antiplatelet therapy using cilostazol and aspirin does not reduce the prevalence of short‐term worsening among patients with acute stroke.

## Appendix

### ADS Investigators

#### Other participating doctors and institutions

Dr Hidetaka Mitsumura (Jikei University School of Medicine), Dr Yuji Ueno (Juntendo University School of Medicine), Dr Masao Watanabe (Juntendo University Urayasu Hospital), Dr Yuki Sakamoto (Nippon Medical School), Dr Shuji Arakawa (Steel Memorial Yawata Hospital), Dr Yoshinari Nagakane (Kyoto Second Red Cross Hospital), Dr Ryota Ishibashi (Kurashiki Central Hospital), Dr Yuka Terasawa, and Dr Koji Fujita (Tokushima University), Dr Kenichi Kashihara (Okayama Kyokuto Hospital), Dr Mutsumi Mitomi and Dr Tatsu Nakano (Yokohama Sakae Kyosai Hospital), Dr Kensaku Shibazaki and Dr Yoshiki Takao (Kurashiki Heisei Hospital), Dr Yohei Tateishi (Nagasaki University) Dr Seiji Goto (Kyushu Medical Center), Dr Yasuhiro Manabe (Okayama Medical Center), Dr Naoaki Kanda (Imamura General Hospital), Dr Toshihiko Ohashi (Seirei Hamamatsu General Hospital), Dr Yoko Okada (Ehime University Graduate School of Medicine), Dr Ryo Itabashi and Dr Eisuke Furui (Kohnan Hospital), Dr Takaaki Takizawa, Dr Masahiro Minami, and Dr Yasuhiro Noguchi (Okayama Tobu Noshinkeigeka Hospital), Dr Yoshiyuki Kondo (Seirei Mikatahara General Hospital), Dr Tesseki Izumi (Nara Medical University), Dr Hirokuni Sakima (University of the Ryukyus), Dr Yasushi Ueno (Shinko Hospital), Dr Junji Kasuya (Atsuchi Neurosurgical Hospital), and Dr Naoki Oba (Heisei Memorial Hospital).

## Sources of Funding

This study received funding from Kawasaki Medical School and Nippon Medical School and an endowment from Otsuka Pharmaceutical Corporation (Tokyo, Japan) that markets cilostazol (Pletaal). Otsuka Pharmaceutical Corporation was blinded to the study design, data collection, and data analysis. This was stated on the informed consent form approved by each institutional review board, and the conflict of interest was appropriately managed at all participating institutions.

## Disclosures

Dr Yamagami reports having received lecture fees and speaker's fees from Otsuka Pharmaceutical, Bayer, and Daiichi‐Sankyo, and a study grant from Bristol‐Myers Squibb. Dr Kenichi Kashihara reports having received lecture fees and speaker's fees from Kyowa Hakko Kirin Co., NOVARTIS, Otsuka Pharmaceutical Co., and Dainippon Sumitomo Pharm Co. Dr Tanaka reports personal fees from honoraria not related to the current work: Bayer Yakuhin, Ltd, Otsuka Pharmaceutical, Co., Ltd, and Sanofi K.K. Dr Kimura reports having received lecture fees and speaker's fees from Otsuka Pharmaceutical Co., Bayer, and Sanofi K.K.

## Supporting information


**Table S1.** Clinical and Laboratory Findings Between the Dual Group and the Aspirin Group
**Table S2.** Imaging Findings Between the Dual Group and the Aspirin Group
**Table S3.** Antiplatelet Therapy and the Concomitant Treatment in the Dual Group and the Aspirin Group
**Table S4.** Clinical Outcome Between the Dual Group and the Aspirin Group
