# Supplementary material for: Acute Aspirin Plus Cilostazol Dual Therapy for Noncardioembolic Stroke Patients Within 48 Hours of Symptom Onset
Source: J Am Heart Assoc. 2019 Jul 26;8(15):e012652. doi: 10.1161/JAHA.119.012652 (PMC6761671; doi:10.1161/JAHA.119.012652)
Supplement: Supplementary file 1 — Table S1. Clinical and Laboratory Findings Between the Dual Group and the Aspirin Group Table S2. Imaging Findings Between the Dual Group and the Aspirin Group Table S3. Antiplatelet Therapy and the Concomitant Treatment in the Dual Group and the Aspirin Group Table S4. Clinical Outcome Between the Dual Group and the Aspirin Group [file JAH3-8-e012652-s001.pdf]

SUPPLEMENTAL MATERIAL

Table S1. Clinical and laboratory findings between the Dual group and the Aspirin group.

Variables	Dual group<br>n=600	Aspirin group<br>n=601	p
Premorbid modified Rankin scale score, median (IQR)	0 (0-0)	0 (0-0)	0.063
Vascular risk factor, n (%)			
Alcohol	275 (46)	274 (46)	0.908
Smoking			0.956
Never	310 (52)	312 (52)	-
Past, beyond 3 months	91 (15)	88 (15)	-
Current	197 (33)	201 (33)	-
Past history, n (%)			
Transient ischemic attack	14 (2)	9 (2)	0.302
Subarachnoid hemorrhage	2 (0.3)	4 (1)	0.687
Heart failure	30 (5)	24 (4)	0.407
Chronic kidney disease			
No	580 (97)	573 (95)	0.220
Without dialysis	17 (3)	24 (4)	-
With dialysis	1 (0.2)	4 (1)	-
Peripheral artery disease	3 (1)	1 (0.2)	0.373
Final diagnosis, n (%)			
No infarction	4 (1)	3 (1)	0.725

Other determined stroke	67 (11)	65 (11)	0.854
Undetermined stroke	55 (9)	64 (11)	0.440
Cardioembolic stroke	22 (4)	15 (3)	0.248
Transient ischemic attack	19 (3)	12 (2)	0.209
Laboratory findings, median (IQR)			
White blood cell count, /l (n=1195)	6720 (5400-8200)	6600 (5400-8200)	0.940
Red blood cell count (n=1195)	457 (420-488)	456 (418-488)	0.484
Hematocrit, % (n=1195)	41.8 (38.7-44.8)	41.8 (38.3-44.8)	0.565
Hemoglobin, mg/dl (n=1195)	14.2 (13.1-15.4)	14.2 (13.0-15.3)	0.421
Platelets, x10 4 μl (n=1196)	21.3 (17.8-25.2)	20.7 (17.3-25.3)	0.287
Aspartate aminotransferase, IU/l (n=1195)	22 (18-27)	23 (18-28)	0.332
Alanine aminotransferase, IU/l (n=1194)	18(14-27)	19 (15-27)	0.667
Albumin, g/dl (n=1164)	4.1 (3.8-4.4)	4.1 (3.8-4.3)	0.322
Lactate dehydrogenase, IU/l (n=1157)	200 (176-233)	202 (174-236)	0.417
Total bilirubin, mg/dl (n=1154)	0.7 (0.5-0.8)	0.7 (0.5-0.9)	0.543
Glucose, mg/dl (n=1182)	118 (102-150)	118 (101-152)	0.885
Blood urea nitrogen, mg/dl (n=1195)	14.0 (11.0-17.0)	14.0 (12.0-18.0)	0.098
Creatinine, mg/dl (n=1196)	0.75 (0.61-0.89)	0.74 (0.61-0.90)	0.983
Total cholesterol, mg/dl (n=1004)	205 (177-233)	199 (176-227)	0.152
Low-density lipoprotein cholesterol, mg/dl (n=1143)	124 (99-147)	119 (97-145)	0.095
High-density lipoprotein cholesterol, mg/dl (n=1153)	49 (41-60)	49 (40-59)	0.612
Triglyceride, mg/dl (n=1159)	115 (85-169)	115 (80-173)	0.620

C-reactive protein, mg/dl (n=1013)	0.15 (0.06-0.31)	0.16 (0.07-0.30)	0.964
D-Dimer, μg/ml (n=1144)	0.7 (0.5-1.2)	0.7 (0.5-1.1)	0.157
Estimate glomerular filtration rate, ml/min./1.73m2 (n=1196)	74.2 (61.1-87.1)	72.8 (61.6-88.0)	0.991
Hemoglobin A1c, % (n=1152)	5.8 (5.4-6.5)	5.8 (5.4-6.6)	0.587
Insulin, μU/ml (n=654)	8.2 (4.8-13.6)	8.6 (4.9-15.1)	0.575
Brain natriuretic peptide, pg/ml (n=996)	25.5 (13.1-52.3)	27.0 (13.4-56.6)	0.195

IQR indicates interquartile range.

Table S2. Imaging findings between the Dual group and the Aspirin group.

Variables	Dual group n=600	Aspirin group n=601	p
Initial MRI			
Infarct territory, n (%)*			
Anterior cerebral artery	18 (3)	16 (3)	0.730
Middle cerebral artery	375 (66)	379 (65)	0.951
Posterior cerebral artery	63 (11)	56 (10)	0.498
Vertebral or basilar artery	130 (23)	139 (24)	0.627
Others	9 (2)	8 (1)	0.812
Infarct location, n (%)*			
Cerebral cortex	103 (18)	105 (18)	1.000
Subcortical white matter	159 (28)	178 (31)	0.300
Basal ganglia (internal capsule)	175 (31)	167 (29)	0.319
Cerebellum	24 (4)	28 (5)	0.671
Thalamus	67 (12)	58 (10)	0.394
Pons	89 (16)	91 (16)	1.000
Others	47 (8)	42 (7)	0.582
Deep and subcortical white matter hyperintensity, Fazekas grade, median (IQR)†	1 (0-2)	1 (0-2)	0.992
Periventricular Hyperintensity, Fukuda grade, median (IQR)‡	1 (0-2)	1 (1-2)	0.275
No microbleeds, n (%)§	424 (71)	434 (73)	0.586
Old intracerebral hemorrhage, n (%)‖	30 (5)	30 (5)	1.000
Symptomatic intracranial stenosis/occlusion on MRA, n (%)‖	139 (23)	133 (22)	0.679

Carotid lesion with ≥50% stenosis or occlusion, n (%)[¶]			
Asymptomatic	22 (4)	26 (4)	0.659
Symptomatic	36 (6)	35 (6)	0.903
Digital subtraction angiography, n (%)**	62 (10)	64 (11)	0.925
Transthoracic echocardiography, n (%)[‖]	510 (85)	487 (81)	0.073
Transesophageal echocardiography, n (%)[‖]	154 (26)	162 (27)	0.646
MRI and MRA on day 7			
infarct[††]			
No lesion	52 (9)	41 (7)	0.281
No change	425 (74)	411 (72)	0.596
Enlargement	71 (12)	87 (15)	0.146
Increment	30 (5)	31 (5)	0.896
Hemorrhagic transformation[‡‡]	11 (2)	16 (3)	0.337
Symptomatic occlusion[§§]	34 (6)	29 (5)	0.605
MRI and MRA on the day of deterioration[‖‖]			
Infarct			
No lesion	1 (2)	1 (2)	1.000
No change	17 (30)	17 (25)	0.553
Enlargement	34 (60)	41 (60)	1.000
Increment	5 (9)	9 (13)	0.572
Hemorrhagic transformation	1 (2)	2 (3)	1.000
Symptomatic occlusion	10 (18)	8 (12)	0.446

*Data on 572 patients in the dual and 580 in the aspirin group were analyzed,[†]597 in the dual and 599 in the aspirin,[‡]597 in the dual and 599 in the dual,[§]597 in the both, [||]597 in the dual and 598 in the aspirin, [¶]594 in the aspirin and 596 in the dual, [**]595 in the dual and 596 in the aspirin, [††]578 in the dual and 570 in the aspirin, [‡‡]574 in the dual and 568 in the aspirin, [§§]577 in the dual and 570 in the aspirin, [||||]57 in the dual and 68 in the aspirin group.

Table S3. Antiplatelet therapy and the concomitant treatment in the Dual group and the Aspirin group.

Variables	Dual group n=600	Aspirin group n=601	p
Assigned antiplatelet therapy for 14 days			
Discontinued drug*			<0.001
Aspirin	2 (3)	39 (87)	-
Cilostazol	25 (31)	1 (2) §	-
Both	53 (66)	5 (11)	-
Dose change of cilostazol†	43 (8)	15 (3)	<0.001
Dose change of aspirin†	26 (5)	47 (9)	0.029
Cilostazol therapy beyond 14 days to 3 months‡			0.061
Dose modification of cilostazol	28 (6)	30 (7)	0.590
Concomitant treatment			
Ozagrel	98 (16)	99 (17)	1.000
Edaravone	504 (85)	511 (85)	0.747
Glycerin	32 (5)	31 (5)	0.898
Low molecular dextran	132 (22)	142 (24)	0.536
Antihypertensive drug			
None	282 (47)	298 (50)	0.386
Angiotensin receptor blocker	242 (41)	242 (41)	1.000
Angiotensin-converting enzyme inhibitor	15 (3)	10 (2)	0.321
Calcium channel blocker	153 (26)	158 (26)	0.792
Beta-antagonist *	17 (3)	21 (4)	0.621

Diuretic drug	18 (3)	11 (2)	0.194
No dyslipidemia drug	241 (40)	249 (42)	0.681
Dyslipidemia drug except statin	23 (4)	15 (3)	0.192
No diabetes drug	440 (74)	435 (73)	0.695
Insulin	47 (8)	35 (6)	0.171
Oral antihyperglycemic drug	128 (22)	147 (25)	0.216

*Data on 81 patients in the dual and 45 in the aspirin group were analyzed, †Data on 514 in the dual and 551 in the aspirin group, ‡data on 470 in the dual and 437 in the aspirin group, §due to early discharge.

Table S4. Clinical outcome between the Dual group and the Aspirin group.

Variables	Dual group n=600	Aspirin group n=601	p
Within 14 days, n (%)*			
Intracerebral hemorrhage and subarachnoid hemorrhage			
Symptomatic	1/2 (50)	0/1 (0)	1.000
Surgery	1/2 (50)	0/1 (0)	1.000
Myocardial infarction	0 (0)	1 (0.2)	1.000
Extracranial hemorrhage			
None	589 (99)	593 (99)	0.752
Adverse events			
None	527 (89)	577 (97)	<0.001
Total hemorrhagic complication	7 (1)	5 (1)	0.578
At discharge, median (interquartile range)†			
Modified Rankin scale score 0	1 (1-2)	1 (1-3)	0.153
From 14 days to 3 months, n (%)			
No recurrence stroke‡	545 (98)	546 (97)	0.419
Ischemic stroke‡	8 (1)	10 (2)	0.813
Transient ischemic attack (TIA)‡	2 (0.4)	3 (1)	1.000
Intracerebral hemorrhage‡	0 (0)	1 (0.2)	1.000
Subarachnoid hemorrhage‡	0 (0)	0 (0)	−
Subdural hematoma‡	0 (0)	1 (0.2)	1.000
Myocardial infarction‡	3 (0.5)	1 (0.2)	0.372

Intracranial hemorrhage‡	0 (0)	2 (0.4)	0.500
Extracranial hemorrhage§			
None	555 (100)	556 (99)	0.031
Gastrointestinal hemorrhage	0 (0)	2 (0.4)	0.500
Urinary hemorrhage	0 (0)	0 (0)	–
Nasal hemorrhage	0 (0)	0 (0)	–
Others	0 (0)	4 (1)	0.124
Serious	0 (0)	2 (0.4)	0.500
Total hemorrhagic complication ‖	0 (0)	8 (1)	0.008
Adverse events ‖			
None	526 (95)	508 (90)	0.009
Gastrointestinal symptoms	3 (1)	2 (0.4)	0.685
Headache	6 (1)	16 (3)	0.050
Palpitation	4 (1)	13 (2)	0.047
Others	17 (3)	23 (4)	0.421
Serious	2 (0.4)	3 (1)	1.000
Major adverse cardiovascular events§	14 (3)	17 (3)	0.716
Within 3 months, n (%)			
Modified Rankin scale score of 0‡	206 (37)	183 (33)	0.117
Major adverse cardiovascular events¶	24 (4)	27 (5)	0.775
Total hemorrhagic complication**	7 (1)	13 (2)	0.259
Intracranial hemorrhage††	2 (0.4)	3 (1)	1.000
Extracranial hemorrhage‡‡			
None	551 (99)	553 (98)	0.299

Gastrointestinal hemorrhage	2 (0.4)	4 (1)	0.687
Urinary hemorrhage	1 (0.2)	0 (0)	0.497
Nasal hemorrhage	0 (0)	0 (0)	−
Others	2 (0.4)	6 (1)	0.287
Serious	2 (0.4)	2 (0.4)	1.000
Adverse events§§			
None	473 (84)	491 (87)	0.147
Gastrointestinal symptoms	5 (1)	2 (0.4)	0.452
Headache	35 (6)	23 (4)	0.137
Palpitation	15 (3)	19 (3)	0.602
Others	35 (6)	28 (5)	0.437
Serious	2 (0.4)	4 (1)	0.687
Neurological deterioration, recurrent ischemic stroke, TIA ||||	74 (13)	76 (13)	1.000

*Data on 594 patients in the dual and 597 in the aspirin group were analyzed, †596 in the dual and 598 in the aspirin, ‡555 in the dual and 561 in the dual, §555 in the dual and 562 in the aspirin, ||556 in the dual and 562 in the aspirin.¶557 in the dual and 563 in the aspirin, **556 in the dual and 562 in the aspirin, ††555 in the dual and 562 in the aspirin, ‡‡556 in the dual and 563 in the aspirin, §§562 in both, ||||559 in the dual and 569 in the aspirin.
